# Editorial Comment: Endoclips as novel fiducial markers in trimodality bladder preserving therapy of muscle-invasive bladder carcinoma: feasibility and patient outcomes

**DOI:** 10.1590/S1677-5538.IBJU.2019.0713.1

**Published:** 2020-11-18

**Authors:** Rodolfo Borges dos Reis, Valdair Francisco Muglia, Antônio Antunes Rodrigues, Gustavo Viani

**Affiliations:** 1 Universidade de São Paulo Faculdade de Medicina de Ribeirão Preto Departamento de Cirurgia e Anatomia Ribeirão PretoSP Brasil Departamento de Cirurgia e Anatomia, Faculdade de Medicina de Ribeirão Preto, Universidade de São Paulo, Ribeirão Preto, SP, Brasil; 2 Universidade de São Paulo Faculdade de Medicina de Ribeirão Preto Departamento de Imagens Médicas, Radioterapia e Oncohematologia Ribeirão Preto Brasil Departamento de Imagens Médicas, Radioterapia e Oncohematologia, Faculdade de Medicina de Ribeirão Preto, Universidade de São Paulo, Ribeirão Preto, Brasil

## COMMENT

Titanium Endoclips (TE) are safe and not expensive. It has been applied in various tissues of the human body due to its high histocompatibility and no foreign body reaction. TE has also been used as a fiducial marker to assist radiotherapy in several tumors, helping the detection of the target area, reducing the damage to healthy tissues (1).

When radiotherapy is planned, the inability to visualize the exact tumor location, due to the bladder wall movement, is by far the biggest challenge to be overcome. The search for the ideal fiducial marker is ongoing in bladder cancer, but today, despite the use of different agents and materials, (2-5) no ideal strategy was found.

The concept to guide radiotherapy in partial bladder treatment opens perspectives for future studies to explore the benefits with its use. Among these benefits, it is noteworthy that partial bladder radiotherapy allows the increase of dose in a reduced volume of the organ based on the rationale of improving the local control, without a significant increase in toxicity (6). Currently, partial bladder radiotherapy is performed using findings from cystoscopy, and pre-surgical tomography (CT) or magnetic resonance (MRI) (7) but The clinical target volume (CTV) delimitation on the planning CT based on pre-surgical images brings uncertainty about the exact tumor boundaries (8). Consequently, to avoid tumor geographic missing, large margins on the CTV to generate the planning tumor volume (PTV) are required (9). The PTV with large volumes limits dose escalation due to the excessive risk of late complications. Thus, the endoscopic bladder fiducials to delimit the CTV give more precision to delineate the target allowing a reduced and more accurate CTV.

Today, there are several forms of image-guided radiotherapy (IGRT) (10). The association between fiducial and IGRT would guarantee more precision to deliver the radiotherapy beam to the target. Among the IGRT system available, in special, IGRT with cone-beam CT combined with bladder fiducials would allow not only a reduction of margins with great precision, but it would also serve as an instrument to control the bladder volume during the radiotherapy fractions, translating in a more significant and realistic relationship between radiotherapy planning and treatment for the prediction of late complications (11). Adaptive radiotherapy combining the cone-beam CT images and treatment planning information still being developed, and in theory, has the potential of improving therapeutic ratio for bladder preservation. In this scenario, the bladder fiducial can have great value to adapt the treatment to the plans (10, 12, 13).

On the other hand, even using simpler IGRT systems, such as planar images from kilovoltage or megavoltage sources, the bladder fiducial also brings precision in controlling the tumor bed's positioning (10). Therefore, the technique developed by Shahbaz et al. has the potential to help for solving historical problems related to organ motion and the inability to safely deliver adequate dose for bladder preservation.

Irritative bladder symptoms are a major source of bothersome for patients, especially when foreign bodies are placed in the bladder. The authors reported no bladder symptoms, or complications as urinary tract infection or hematuria, during the follow-up, but we need to take into account that no symptom severity scale was applied. The small sample size and the short follow-up do not support any treatment effectiveness conclusion. We congratulate the authors by their innovative approach in a challenge field (14).
